# Risk and protective factors for SARS-CoV-2 reinfections, surveillance data, Italy, August 2021 to March 2022

**DOI:** 10.2807/1560-7917.ES.2022.27.20.2200372

**Published:** 2022-05-19

**Authors:** Chiara Sacco, Daniele Petrone, Martina Del Manso, Alberto Mateo-Urdiales, Massimo Fabiani, Marco Bressi, Antonino Bella, Patrizio Pezzotti, Maria Cristina Rota, Flavia Riccardo, on behalf of the Italian Integrated Surveillance of COVID-19 study group

**Affiliations:** 1 Department of Infectious Diseases, Istituto Superiore di Sanità, Rome, Italy; 2 The members of the Italian Integrated Surveillance of COVID-19 study group are acknowledged at the end of the article

**Keywords:** SARS-CoV-2 reinfections, severe disease, risk factors, Omicron variant, Italy

## Abstract

We explored the risk factors associated with SARS-CoV-2 reinfections in Italy between August 2021 and March 2022. Regardless of the prevalent virus variant, being unvaccinated was the most relevant risk factor for reinfection. The risk of reinfection increased almost 18-fold following emergence of the Omicron variant compared with Delta. A severe first SARS-CoV-2 infection and age over 60 years were significant risk factors for severe reinfection.

Between the end of 2021 and the beginning of 2022, the severe acute respiratory syndrome coronavirus 2 (SARS-CoV-2) Omicron variant (Phylogenetic Assignment of Named Global Outbreak (Pango) lineage designation B.1.1.529) emerged in Europe. Its high immune escape potential [1], highlighted by reduced vaccine effectiveness [2] and by an increase in reinfections [3], is thought to explain, at least partly, its increased transmissibility [4,5].

In this study, we explored the incidence and risk factors associated with SARS-CoV-2 reinfections, both overall and severe, in Italy during the time in which dominance shifted from the Delta (B.1.617.2) to the Omicron variant, in order to better understand how the SARS-CoV-2 epidemic changed in Italy.

## Data source and outcome definition

Reinfections in Italy are defined as a laboratory-confirmed SARS-CoV-2 infection occurring ≥ 90 days (≥ 60 days if genotyping results showing different variants is available) after the onset of the previous laboratory-confirmed infection and have been under surveillance since 24 August 2021 [6].

Data were obtained using deterministic record linkage by individual tax code, combining data from the Italian National coronavirus disease (COVID-19) Integrated Surveillance System on SARS-CoV-2 infections coordinated by the Italian National Institute of Health [7], with data from the National Vaccination Registry coordinated by the Italian Ministry of Health [8], both updated on 9 March 2022. We considered in our analysis only the individuals with at most one episode of reinfection, excluding all individuals who reported more than one reinfection (less than 0.01% among all the notified reinfections).

**Figure 1:**
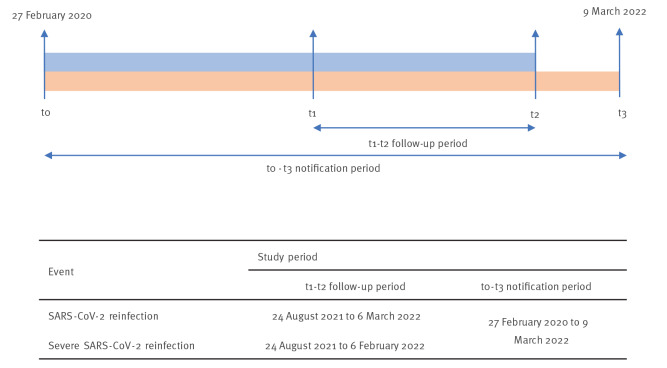
Timeline of periods of selection and events in the study population, SARS-CoV-2 reinfections, Italy, August 2021–March 2022

We considered two endpoints: SARS-CoV-2 reinfections and severe SARS-CoV-2 reinfections (SARS-CoV-2 reinfection with subsequent admission to hospital or death within 28 days). The timeline periods for each event are shown in Figure 1 (further details on the study period are given in the Supplement, section 1). In order to account for the longer disease progression time, the study period for the estimation of the risk of severe SARS-CoV-2 disease was considered up to 6 February 2022. This allowed us to consider all notified cases of confirmed infection with a follow-up period of at least 28 days, to document possible worsening of clinical symptoms and the possible delay in notification.

SARS-CoV-2: severe acute respiratory syndrome coronavirus 2.

The blue bar refers to the period when participants could be diagnosed from primary infection (t_0_–t_2_) or reinfection (t_1_–t_2_). The orange bar refers to the notification period.

In the study period (24 Aug 2021–6 Mar 2022), Italy experienced the dominant transmission of two different SARS-CoV-2 variants of concern (VOC) [9]. The Delta variant was dominant from 24 August 2021 to 5 December 2021, the Omicron variant became dominant from 3 January 2022 [10], with an intermediate period in which prevalence transitioned from Delta to Omicron (6 Dec 2021–2 Jan 2022). The Omicron BA.1 sublineage was prevalently circulating during the study period. BA.2 was first detected in January 2022 but became dominant only in April [11].

## Estimation of the risk of any reinfection

We estimated the incidence rate ratios (RR) of any SARS-CoV-2 reinfections using the generalised linear mixed model, assuming a negative binomial distribution, since we verified that our data are significantly over-dispersed. We considered as random effect the geographical region of diagnosis, as offset the time of exposure measured in days, and adjusting for the regional weekly incidence in the general population. We included as time-dependent fixed covariates the age group (0–19, 20–39, 40–59, 60–79 and ≥ 80 years) and the vaccination status. Based on previous studies, we considered participants as vaccinated 14 days after inoculation of the vaccine. Thus, participants were classified – on a daily basis – as unvaccinated (never received a dose or 0–14 days from first dose), vaccinated (with at least one dose) for 120 days or less, and vaccinated (with at least one dose) for more than 120 days. The time-invariant covariates included in the model as fixed effects were: VOC predominance phase (Delta phase, transition phase, Omicron phase), number of days elapsed from first diagnosis (90–210, 211–330, 331–450, > 450), sex (male/female), healthcare worker status (yes/no), and nationality (Italian/non-Italian).

In the study period, 8,413,857 cases of SARS-CoV-2 infection were notified in Italy, including 249,121 cases of documented SARS-CoV-2 reinfection. 

**Figure 2:**
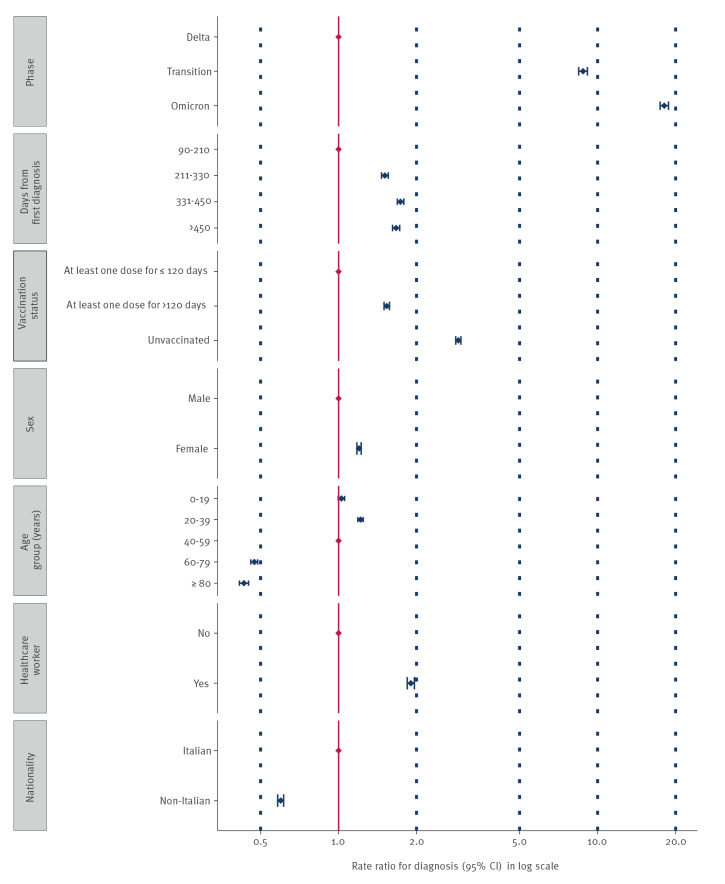
Forest plot of adjusted incidence rate ratios (in log scale) for all SARS-CoV-2 reinfections by epidemic phase, Italy, 24 August 2021 to 6 March 2022 (n =249,121^a^)

CI: confidence interval; SARS-CoV-2: severe acute respiratory syndrome coronavirus 2.

^a^ See Figure 1 and the Supplement, section 1 for the inclusion criteria. The number of reinfection depends on the study period. To estimate the risk of any reinfection we considered a study period that ranged from 24 August 2021 to 6 March 2022. In order to account for the disease progression within 28 days since reinfection, the study period for the estimation of the risk of SARS-CoV-2 reinfection leading to hospitalisation or death (severe reinfection) was censored on 6 February 2022. From 24 August 2021 to 6 March 2022, 249,121 reinfections were notified, whereas 206,013 is the number of reinfections notified from 24 August 2021 to 6 February 2022.

Compared with the Delta phase, the adjusted risk of reinfection during the Omicron phase in Italy was 18.1 times higher (95% confidence interval (CI): 17.4–18.8) (Figure 2, Table 1). Regardless of the predominating VOC, compared with persons who were vaccinated (with at least one dose) for ≤120 days, the risks of reinfection among unvaccinated people and among those who were vaccinated (with at least one dose) for more than 120 days were 2.9 (95% CI: 2.8–3.0) and 1.5 (95% CI: 1.5–1.6) times higher, respectively.

Delta phase: 24 Aug–5 Dec 2021; transition phase: 6 Dec 2021–2 Jan 2022; Omicron phase: 3 Jan–6 Mar 2022. The incidence rate ratios are adjusted also for standardised weekly regional incidence. Red line: rate ratio for the reference class.

**Table 1:** Adjusted incidence rate ratios of SARS-CoV-2 reinfection, Italy, 24 August 2021–6 March 2022 (n =249,121)

		Reinfection (249,121)	PD (842,254,906)	Incidence per 100,000 PD	RR adjusted (95% CI)	p value
Epidemic phase^a^	Delta	6,028	434,476,324	1.39	Reference	
	Transition	36,509	124,883,250	29.23	8.79 (8.45–9.13)	< 0.001
	Omicron	206,584	282,895,332	73.02	18.09 (17.43–18.77)	< 0.001
Days from first diagnosis	90–210	25,126	147,300,836	17.06	Reference	
	210–330	67,149	319,935,680	20.99	1.51 (1.47–1.56)	< 0.001
	330–450	121,934	284,817,183	42.81	1.73 (1.69–1.79)	< 0.001
	> 450	34,912	90,201,207	38.70	1.67 (1.62–1.72)	< 0.001
Vaccination status	Vaccinated with at least one dose for ≤ 120 days	81,235	408,702,801	19.88	Reference	
	Vaccinated with at least one dose for > 120 days	77,829	216,463,056	35.95	1.53 (1.5–1.57)	< 0.001
	Unvaccinated	90,057	217,089,049	41.48	2.90 (2.83–2.97)	< 0.001
Sex	Male	112,804	410,792,861	27.46	Reference	
	Female	136,317	431,462,045	31.59	1.20 (1.18–1.22)	< 0.001
Age group (years)	0–19	51,212	129,028,476	39.69	1.03 (1.00–1.06)	0.0806
	20–39	82,037	211,577,554	38.77	1.22 (1.19–1.25)	< 0.001
	40–59	85,978	283,687,021	30.31	Reference	
	60–79	21,268	160,343,346	13.26	0.47 (0.46–0.49)	< 0.001
	≥ 80	8,626	57,618,509	14.97	0.43 (0.41–0.45)	< 0.001
Nationality	Italian	227,273	767,676,929	29.61	Reference	
	Foreign	21,848	74,577,977	29.30	0.60 (0.58–0.61)	< 0.001
Healthcare worker	No	236,178	815,250,169	28.97	Reference	
	Yes	12,943	27,004,737	47.93	1.90 (1.84–1.96)	< 0.001

CI: confidence interval; IRR: incidence rate ratios; PD: person-days; SARS-CoV-2: severe acute respiratory syndrome coronavirus 2.

^a^ Epidemic phases: Delta: 24 Aug–5 Dec 2021; transition: 6 Dec 2021–2 Jan 2022; Omicron: 3 Jan–6 Mar 2022.

The adjusted incidence rate ratio (RR) for reinfection increased progressively during the study period, being highest during the Omicron phase, across all age groups (see the Supplement, section 2, for a detailed breakdown by epidemic phase, vaccination status and age group). Lower RR were observed among people older than 60 years, who also had the highest vaccination coverages, both overall and separately for each epidemic phase (Table 1 and Figure 2).

## Estimation of the risk of severe SARS-CoV-2 reinfection (hospitalisation or death within 28 days from diagnosis)

Among all reinfected cases we estimated the cumulative incidence risk ratio (IRR) of hospitalisation or death within 28 days from diagnosis of SARS-CoV-2 reinfection using the negative binomial generalised linear mixed model including as random effect the geographical region of diagnosis and adjusting for the regional weekly incidence in the general population. In this analysis all included cases were considered exposed for the same time-window period. We considered as fixed risk factors of severe SARS-CoV-2 reinfections VOC predominance phase, severity of first SARS-CoV-2 infection (hospitalisation within 28 days from diagnosis), vaccination status (at the time of reinfection diagnosis), coded as unvaccinated (never received a dose or with a diagnosis of reinfection within 14 days from the first dose) and vaccinated with at least one dose (with a diagnosis of reinfection at least 14 days from the last dose), age group, sex, healthcare worker status and nationality.

From 24 August 2021 to 6 February 2022, of 206,013 documented reinfections which occurred ≥ 90 days after the first reported infection, 2,139 were reported as severe.

The adjusted risk of a reinfected case being reported with severe COVID-19 was significantly higher among persons with a severe first SARS-CoV-2 infection, among those 60 years or older and among non-Italian nationals (Table 2). Both age and first infection severity are predictors of severe reinfection (Section 3 of the Supplement contains an analysis of the relation between these two variables). We observed an effect of the dominant SARS-CoV-2 variant, with a progressively lower risk of severe reinfection from the Delta phase to the transition and Omicron phases.

**Table 2:** Adjusted cumulative incidence risk ratios for severe SARS-CoV-2 within 28 days since reinfection, Italy, 24 August 2021–6 February 2022 (n = 206,013^a^)

		Non-severe reinfection (203,874)		Severe reinfection (2,139)		Cumulative Incidence per 100,000	IRR adjusted (95% CI)	p value
		n	%	n	%			
Epidemic phase^b^	Delta	5,685	3	345	16	5,721	Reference	
	Transition	36,111	18	404	19	1,106	0.47 (0.39–0.57)	< 0.001
	Omicron	162,078	79	1,390	65	850	0.37 (0.3–0.46)	< 0.001
Severity first diagnosis	No	195,921	96	1,584	74	802	Reference	
	Yes	7,953	4	555	26	6,523	2.86 (2.55–3.22)	< 0.001
Vaccination status	At least one dose	131,424	64	1,545	72	1,162	Reference	
	Unvaccinated	72,450	36	594	28	813	1.07 (0.96–1.19)	0.243
Sex	Male	93,239	46	963	45	1,022	Reference	
	Female	110,635	54	1,176	55	1,052	0.95 (0.86–1.05)	0.285
Age group (years)	0–19	41,597	20	124	6	297	0.55 (0.44–0.68)	< 0.001
	20–39	69,717	34	378	18	539	0.98 (0.84–1.15)	0.768
	40–59	70,516	35	399	19	563	Reference	
	60–79	16,513	8	643	30	3,748	5.45 (4.72–6.28)	< 0.001
	≥ 80	5,531	3	595	28	9,713	13.09 (11.21–15.29)	< 0.001
Healthcare worker	No	193,551	95	2,050	96	1,048	Reference	
	Yes	10,323	5	89	4	855	1.03 (0.82–1.29)	0.800
Nationality	Italian	185,573	91	1,903	89	1,015	Reference	
	Non-Italian	18,301	9	236	11	1,273	1.68 (1.45–1.96)	< 0.001

^a ^See Figure 1 and the Supplement, section 1 for the inclusion criteria. The number of reinfection depends on the study period. To estimate the risk of any reinfection we considered a study period that ranged from 24 August 2021 to 6 March 2022. In order to account for the disease progression within 28 days since reinfection, the study period for the estimation of the risk of SARS-CoV-2 reinfection leading to hospitalisation or death (severe reinfection) was censored on 6 February 2022. From 24 August 2021 to 6 March 2022, 249,121 reinfections were notified, whereas 206,013 is the number of reinfections notified from 24 August 2021 to 6 February 2022.

^b^ Epidemic phases: Delta: 24 Aug–5 Dec 2021; transition: 6 Dec 2021–2 Jan 2022; Omicron: 3 Jan–6 Feb 2022.

## Discussion

In Italy, we found a 18-fold increase in the risk of reinfection during the Omicron (sublineage BA.1) phase compared with previous epidemic phases characterised by the predominance of the Delta variant or a mix of Delta and Omicron variants. By contrast, the risk of severe reinfection leading to hospitalisation or death appeared reduced during the period with predominant circulation of the Omicron variant. Our findings are consistent with those from other studies suggesting that the Omicron variant can better evade prior immunity [12,13] but is less frequently associated with severe clinical outcomes [14,15].

Prior severe infection has emerged as an independent risk factor for severe reinfection, which could be interpreted as a proxy of increased vulnerability (e.g. because of pre-existing diseases). While this suggests that patient vulnerability and age are both predictors of a risk of severe reinfection, we are unable with our findings to disentangle the role of age alone from patient vulnerability (which tends to increase with age) or speculate whether increased vulnerability is a better predictor of severe reinfection than age alone.

Vaccination was identified as a protective factor against SARS-CoV-2 reinfection, particularly for those vaccinated with at least one dose within 120 days. Although older age (≥ 60 years) was a risk factor for severe disease, the high vaccination coverage achieved in this age group played a protective role against the risk of any reinfection.

We also observed a reduced risk of any SARS-CoV-2 reinfection but a higher risk of developing a severe reinfection in non-Italian nationals compared with Italian nationals. As suggested by a previous study conducted in Italy, underdiagnosis and delayed diagnosis could partly explain the lower incidence, but worse clinical outcomes are observed in this population group [16].

Our study has several limitations. Firstly, the observation time for severe infections in the most recent Omicron phase is short compared with that for the Delta phase. However, we think this has not greatly affected the precision of our estimates because, despite the shorter follow-up time, a very large number of infections occurred in Italy between December 2021 and January 2022 compared with previous periods. Secondly, since our data did not include information on possible deaths that occurred for causes unrelated to COVID-19, it is likely that the risk of reinfection is underestimated for people older than 60 years. Finally, we were unable to adjust for co-morbidities (e.g. immune disorders) and therapies (e.g. monoclonal antibodies, antiviral drugs), factors that could affect the characteristics or duration of the natural or acquired immune response.

## Conclusion

Our findings show a risk of SARS-CoV-2 reinfection 18-fold higher for the Omicron than for Delta variant. Regardless of the variant, being unvaccinated was the most relevant risk factor for reinfection. The most predictive factors for the risk of hospitalisation or death within 28 days from diagnosis of reinfection were age and the disease severity at first episode.

## Supplementary Data

159000 Supplement
