# Supplementary material for: Risk and protective factors for SARS-CoV-2 reinfections, surveillance data, Italy, August 2021 to March 2022
Source: Euro Surveill. 2022 May 19;27(20):2200372. doi: 10.2807/1560-7917.ES.2022.27.20.2200372 (PMC9121659; doi:10.2807/1560-7917.ES.2022.27.20.2200372)
Supplement: Supplement [file 22-00372_SACCO_Supplement.pdf]

Supplementary materials to the article:

Risk and protective factors for SARS-CoV-2 reinfections, surveillance data, Italy, August 2021 to March 2022

This supplementary material is hosted by Eurosurveillance as supporting information alongside the article 'Risk and protective factors for SARS-CoV-2 reinfections, surveillance data, Italy, August 2021 to March 2022', on behalf of the authors, who remain responsible for the accuracy and appropriateness of the content. The same standards for ethics, copyright, attributions and permissions as for the article apply. Supplements are not edited by Eurosurveillance and the journal is not responsible for the maintenance of any links or email addresses provided therein.

Supplementary Material 1

To estimate the risk of SARS-CoV-2 reinfection, our study cohort included all subjects with a confirmed SARS-CoV-2 infection (first positive SARS-CoV-2 test), in Italy between 27th February 2020 to 22nd November 2021, who were still alive on 24th August 2021. The exit date for all the subjects was the date of reinfection, the date of death or the end of follow-up on 6th March 2022, whichever came first. The study period was from the 24th August 2021 to the 6th March 2022.

In order to account for the disease progression time, the study period for the estimation of the risk of SARS-CoV-2 reinfection leading to hospitalisation or death (severe reinfection) was censored on 6th February 2022. This allowed us to consider all notified cases of confirmed reinfection with an additional 28-day observation time available to ascertain possible worsening of clinical symptoms, accounting for three days of possible delay in notification. To assess the risk of severe SARS-CoV-2 reinfections we therefore included in the analysis all persons with a documented reinfection from 24th August 2021 to 6th February 2022.

Supplementary Material 2

Table S1. Adjusted incidence risk ratios for all SARS-CoV-2 reinfections (24 August 2021 to 6 March 2022) by epidemic phase, vaccination status and age group, and vaccination coverage by epidemic phase, Italy

Vaccination status and coverage by epidemic phase		Age group				
		**0-19**	**20-39**	**40-59**	**60-79**	**80+**
**Epidemic phase[a]: Delta**						
IRR	*At least one dose from ≤120 days*	1.03 (1-1.06)	1.22 (1.19-1.25)	*ref.*	0.47 (0.46-0.49)	0.43 (0.41-0.45)
	At least one dose from >120 days	1.57 (1.52-1.63)	1.87 (1.8-1.93)	1.53 (1.5-1.57)	0.73 (0.7-0.75)	0.66 (0.63-0.69)
	Unvaccinated	2.97 (2.87-3.08)	3.53 (3.41-3.65)	2.9 (2.83-2.97)	1.37 (1.32-1.43)	1.25 (1.19-1.31)
Coverage primary as of 5th December 2021 (%)		31.4	83.5	84.9	91.2	95.7
Coverage booster as of 5th December 2021 (%)		0.2	5.7	12.0	24.4	58.0
**Epidemic phase[a]: Transition**						
IRR	*At least one dose from ≤ 120 days*	9.01 (8.59-9.01)	10.69 (10.21-10.69)	8.79 (8.45-8.79)	4.15 (3.96-4.15)	3.79 (3.58-3.79)
	At least one dose from > 120 days	13.83 (13.11-13.83)	16.4 (15.58-16.4)	13.48 (12.88-13.48)	6.37 (6.04-6.37)	5.82 (5.47-5.82)
	Unvaccinated	26.13 (24.78-26.13)	30.99 (29.42-30.99)	25.48 (24.33-25.48)	12.05 (11.4-12.05)	10.99 (10.32-10.99)
Coverage primary as of 3rd January 2022 (%)		33.2	85.1	86.0	91.8	96.1
Coverage booster as of 3rd January 2022 (%)		1.9	20.1	36.5	58.1	75.0
**Epidemic phase[a]: Omicron**						
IRR	*At least one dose from ≤ 120 days*	18.56 (17.72-19.43)	22 (21.05-23)	18.09 (17.43-18.77)	8.55 (8.16-8.97)	7.8 (7.39-8.24)
	At least one dose from > 120 days	28.47 (27.01-30.01)	33.76 (32.09-35.52)	27.76 (26.55-29.02)	13.13 (12.44-13.85)	11.97 (11.27-12.72)
	Unvaccinated	53.81 (51.09-56.66)	63.8 (60.63-67.15)	52.46 (50.18-54.85)	24.8 (23.51-26.17)	22.63 (21.3-24.04)
Coverage primary as of 6th March 2022 (%)		48.0	88.8	88.9	94.2	97.7
Coverage booster as of 6th March 2022 (%)		17.7	60.2	71.1	85.2	88.4

[a] Epidemic phase: Delta from 24/08/2021 to 05/12/2021; Transition from 06/12/2021 to 02/01/2022; Omicron from 03/01/2022 to 06/03/2022

Supplementary Material 3

In the table below (Table S2) it has been reported the adjusted incidence rate ratios (IRR) of severe SARS-CoV-2 first infection for the age groups. We can observe that older age (≥60 years) resulted to be a predictive risk factor for severe first infection, too.

Table S2. Adjusted incidence risk ratios of severe SARS-CoV-2 first infection, Italy, 24 August 2021 to 6 February 2022.

		**IRR (CI 95%)**
**Age group**	0-19	0.21 (0.20-0.22)
	20-39	0.56 (0.53-0.58)
	40-59	*ref.*
	60-79	4.41 (4.24-4.58)
	80+	15.8 (15.1-16.4)

Focusing on the risk of severe SARS-CoV-2 reinfections, we estimated the impact of severity of first infection and age group on the risk of severe SARS-CoV-2 reinfections using the Negative Binomial Generalized Linear Mixed Model including as random effect the geographical region of diagnosis and adjusting for VoC predominance phase, vaccination status, sex, healthcare worker status, nationality and the weekly regional incidence. To analyse the effect of the age and the severity of first infection on the risk of severe SARS-CoV-2 three different model were estimated: we included only the severity of first infections as a fixed effect in Model 1, only the age group in Model 2 and both variables in Model 3 (the model proposed in the manuscript, Table 2).

In the table below (Table S3) we reported the IRRs of severe SARS-CoV-2 reinfections estimated in correspondence of three estimated models. Model 3 resulted as the best model in terms of AIC (Akaike Information Criterion) and BIC (Bayesian Information Criterion), highlighting that both variables concur to explain the risk of severe reinfection. We can observe that first infection severity modulates the effect of age by decreasing the IRRs for those aged 60-79 to 80+ years.

Table S3. Comparison of adjusted incidence risk ratios of severe SARS-CoV-2 reinfection (Italy, 24 August 2021 to 6 February 2022).

		**Model 1**	**Model 2**	**Model 3**
**Severity first diagnosis**	No	*ref.*	-	*ref.*
	Yes	4.97 (4.3-5.75)	-	2.86 (2.55-3.22)
**Age group**	0-19	-	0.49 (0.39-0.61)	0.55 (0.44-0.68)
	20-39	-	0.92 (0.78-1.08)	0.98 (0.84-1.15)
	40-59	-	*ref.*	*ref.*
	60-79	-	6.41 (5.52-7.44)	5.45 (4.72-6.28)
	80+	-	16.61 (14.12-19.53)	13.09 (11.21-15.29)
	**AIC**	8922	7923	7654
	**BIC**	9001	8022	7761
